# Supplementary material for: Diversifying crop rotation increases food production, reduces net greenhouse gas emissions and improves soil health
Source: Nat Commun. 2024 Jan 3;15:198. doi: 10.1038/s41467-023-44464-9 (PMC10764956; doi:10.1038/s41467-023-44464-9)
Supplement: Supplementary file 1 — Clean Supplementary Information [file 41467_2023_44464_MOESM1_ESM.docx]

***Supplementary Materials***

**Diversifying crop rotation increases food production, reduces net greenhouse gas emissions and improving soil health**

Xiaolin Yang^1,2*^, Jinran Xiong^1,2^, Taisheng Du^1,2*^, Xiaotang Ju^3*^, Yantai Gan^4,5*^, Sien Li^1,2^, Longlong Xia^6^, Yanjun Shen^7^, Steven Pacenka^8^, Tammo S. Steenhuis^8^, Kadambot H.M. Siddique^9^, Shaozhong Kang^1,2^, Klaus Butterbach-Bahl^10,11^

*1. State Key Laboratory of Efficient Utilization of Agricultral Water Resources, Beijing 100083, China*

*2. College of Water Resources & Civil Engineering, China Agricultural University, Beijing, 100083, People’s Republic of China*

*3. School of Tropical Agriculture and Forestry, Hainan University, Haikou 570228, China*

*4. College of Life and Environmental Sciences, Wenzhou University, Wenzhou, Zhejiang, 325035, China;*

*5. The μBC-Soil Group, Tallus Heights, Kelowna, BC, Canada*

*6.* *State Key Laboratory of Soil and Sustainable Agriculture, Institute of Soil Science, Chinese Academy of Sciences, Nanjing 210008, China.*

*7.* *Key Laboratory of Agricultural Water Resources, Centre for Agri-cultural Resources Research, Institute of Genetics and Develop-mental Biology, Chinese Academy of Sciences, Shijiazhuang, 050022, China*

*8. Department of Biological and Environmental Engineering, Riley-Robb Hall, Cornell University, Ithaca, NY, 14853, United States of America*

*9. The UWA Institute of Agriculture, The University of Western Australia, Perth, WA 6001, Australia*

*10. Land-CRAFT, Department of Agroecology, Aarhus University, Aarhus, Denmark*

*11. Institute of Meteorology and Climate Research, Atmospheric Environmental Research (IMK-IFU), Karlsruhe Institute of Technology (KIT),* Garmisch Partenkirchen*, Germany*

^*^ Corresponding author(s):

Xiaolin Yang, Email: yangxiaolin429@cau.edu.cn

Taisheng Du, E-mail: dutaisheng@cau.edu.cn

Xiaotang Ju, E-mail: juxt@cau.edu.cn

Gary Y. Gan, Email: [gary.gan@wzu.edu.cn](mailto:gary.gan@ubc-soil.ca)

**Supplementary Methods**

**Nutrition score**

In addition to harvest weight and volume, we also determined the nutritional value of harvested crops based on their nutrient composition[^1^](#_ENREF_1) (Table S3). We extracted 17 nutrient concentrations for each crop—N, P, K, protein, fat, magnesium, calcium, sodium, iron, zinc, vitamin A, thiamin, riboflavin, folate, niacin, vitamin B6, and vitamin E—which we summarized as a nutrition score using the entropy weight method[^2^](#_ENREF_2). The entropy weight method uses the weight value of each nutrient in categories of ‘good and bad’ to calculate the comprehensive nutritional score of each crop as a weighted average of its nutrients:

(1) Data standardization

$y_{ij}=\frac{X_{ij}-X_{\min}}{X_{\max}-X_{\min}}$ (1)

where *y_ij_* is the standardized value of each indicator, *X_min_* is the minimum value of the indicators, and *X_max_* is the maximum value of the indicators, with *n* samples and *k* indicators, and *X_ij_* is the value of the *j*th indicator and *i*th sample (*i* = 1, 2, ..., n; *j* = 1, 2, …, k).

(2) Information entropy

$E_{j}=-\ln(n)^{-1}\sum_{i=1}^{n} p_{ij}\ln p_{ij}$ (2)

$p_{ij}=\frac{y_{ij}}{\sum_{i=1}^{n} y_{ij}}$ (3)

where *E_j_* is the information entropy of each indicator value. When *p_ij_* = 0, ln*p_ij_* was modified as follows:

$p_{ij}=\left(1+y_{ij}\right)/\sum_{j=1}^{m} \left(1+y_{ij}\right)$ (4)

(3) Calculating the entropy weight (*W_j_*) of the evaluated indicator:

$W_{j}=\frac{1-\text{E}_{\text{j}}}{\text{n}-\sum_{i=1}^{n} \text{E}_{\text{i}}}$ (5)

4) Calculating the entropy comprehensive index for nutrition score:

$Z_{i}=\sum_{j=1}^{k} p_{ij}W_{j}$ (6)

**Soil biodiversity**

We calculated the alpha diversity index[^3^](#_ENREF_3) to reflect the soil microbial biodiversity in each treatment. The alpha diversity index combines the results of six indices: Shannon-Weaver, Chao1, ACE, Richness, Simpson, and Pielou indices[^4^](#_ENREF_4)^,^[^5^](#_ENREF_5), calculated using OTU numbers for bacteria and fungi:

$Observed\text{ }richness=S_{obs}$ (7)

where *S_obs_* is the observed OTU number, detailed in Additional Methods (soil microbial diversity).

$Chao1=S_{obs}+\frac{S_{n_{1}}^{2}}{2S_{n_{2}}}$ (8)

where *Chao1* is the abundance-based estimator of species, *Sn_1_* is the OTU number with singletons, and *Sn_2_* is the OTU number with doubletons.

$ACE=S_{abund}+\frac{S_{\text{r}are}}{C_{ACE}}+\frac{S_{n_{1}}}{C_{ACE}}\gamma_{ACE}^{2}$ (9)

where *ACE* is the abundance-based coverage estimator of species richness, *S_abund_* is the number of OTUs with absolute abundance above 10, *S_rare_* is the number of OTUs with absolute abundance below 10, and *C_ACE_* is defined as:

$C_{ACE}=1-\frac{S_{n_{1}}}{N_{rare}}$ (10)

where *N_rare_* is the total number of sequences in the rare OTUs and γ^2^_ACE_ is defined as:

$\gamma_{ACE}^{2}=\max\left[\frac{S_{rare}}{C_{ACE}}\frac{i\left(i-1\right)S_{n_{1}}}{N_{rare}\left(N_{rare}-1\right)}-1,0\right]$ (11)

$Shannon\text{ }Index=H'=-\sum_{i=1}^{S_{\text{obs}}} \frac{n_{i}}{N}\ln\frac{n_{i}}{N}$ (12)

where *H’* is the Shannon index (estimator of species richness and species evenness: more weighting on species richness), *n_i_* is the number with sequence *I*, and N is the number of all sequences.

$Simpson=1-\frac{\sum_{i=1}^{S_{obs}} n_{i}\left(n_{i}-1\right)}{N\left(N-1\right)}$ (17)

$Pielou=\frac{H'}{\ln\left(S_{obs}\right)}$ (18)

where the Simpson index is an estimator of species richness and species evenness (more weighting on species evenness) and Pielou is an estimator of species evenness.

***Supplementary Results Figures***

**Fig. S1** Time series of at least weekly measured soil N_2_O fluxes as observed for the different crop rotations. Values are means (*n*=3) ± SE for each treatment. Error bar for each dot is the standard deviation of three replicates. Treatment abbreviations: WM: winter wheat–summer maize (Control); SpWM: sweet potato→winter wheat–summer maize rotation; PWM: peanut→winter wheat–summer maize rotation, SWM: soybean→winter wheat–summer maize rotation, SmWM: spring maize→winter wheat–summer maize rotation, RSWM: ryegrass–sorghum→winter wheat–summer maize rotation.

**Fig. S2** Time series of at least weekly measured soil CH_4_ fluxes as observed for the different crop rotations. Values are means (*n*=3) ± SE for each treatment. Error bar for each dot is the standard deviation of three replicates. Treatment abbreviations: WM: winter wheat–summer maize (Control); SpWM: sweet potato→winter wheat–summer maize rotation; PWM: peanut→winter wheat–summer maize rotation, SWM: soybean→winter wheat–summer maize rotation, SmWM: spring maize→winter wheat–summer maize rotation, RSWM: ryegrass–sorghum→winter wheat–summer maize rotation.

**Fig. S3** Variations in soil physicochemical and biological properties with different crop rotations in 2016 and 2022. Values are the mean of three replicates samples taken in October 2016 or October 2022. Different lowercase letters indicate significant differences between crop rotations for each indicator at *P* < 0.05, respectively. SOC: soil organic carbon, TN: total N, MBC: microbial biomass carbon, MBN: microbial biomass N, DOC: dissolved organic carbon, AP: available P, BD: bulk density, and SWC: soil water content. Treatment abbreviations: WM: winter wheat–summer maize (Control); SpWM: sweet potato→winter wheat–summer maize rotation; PWM: peanut→winter wheat–summer maize rotation, SWM: soybean→winter wheat–summer maize rotation, SmWM: spring maize→winter wheat–summer maize rotation, RSWM: ryegrass–sorghum→winter wheat–summer maize rotation.

**Fig. S4** Changes in soil microbial biodiversity indexes between sampling dates in 2016 and 2022 following the implementation of six diversified crop rotations. (a–f): bacteria; (g–l): fungi. See SI Methods for the index calculations (Shannon Index, Chao 1, OTU Richness, ACE index, Pielou, and Simpson). Values are the mean of three replicates samples taken in October 2016 or October 2022. Different lowercase letters indicate significant differences between crop rotations for each indicator at *P*< 0.05, respectively. Treatment abbreviations: WM: winter wheat–summer maize (Control); SpWM: sweet potato→winter wheat–summer maize rotation; PWM: peanut→winter wheat–summer maize rotation, SWM: soybean→winter wheat–summer maize rotation, SmWM: spring maize→winter wheat–summer maize rotation, RSWM: ryegrass–sorghum→winter wheat–summer maize rotation.

***Supplementary Methods Figures***

**Fig. S5** Location of Luancheng experimental station (a), annual precipitation from 1960 to 2022 (b), and monthly precipitation and average daily air temperature from 2016 to 2022 (c). (a) made using ArcGIS (Ver.10.5, ESRI Inc., CA, USA, 2016).

**Fig. S6** Graphical outline of cropping sequences and timing of field operations for the six investigated crop rotations (a). See Supplementary Data 1 for dates, irrigation amounts, and fertilizer rates. Photos of winter wheat (b), summer maize (c), sweet potato (d), peanuts (e), soybean (f), spring maize (g), ryegrass (h), and sorghum (i).

**Fig. S7** System boundary for calculating net greenhouse emissions in different crop rotations. (The small objects generated using BioRender (https://biorender.com/)).

**Fig. S8** Scree plot of eigenvalues against the number of principal components (a, b) for soil indicators from six crop rotations.

**Table S1** Annual agricultural inputs for each crop in six crop rotations from 2016 to 2022

**Table S2** CPI, price, and cost per hectare of diverse crops from 2016 to 2022

**Table S3** Nutritional content of different crops as reported in the literature

**Table S4** Emission coefficients of carbon dioxide equivalent for agricultural inputs as reported in the literature

**Table S5** Weighting factors based on principal component analysis of eigen-vectors for the different soil indicators to calculate the soil health score for different crop rotations

**Supplementary Results Figures**


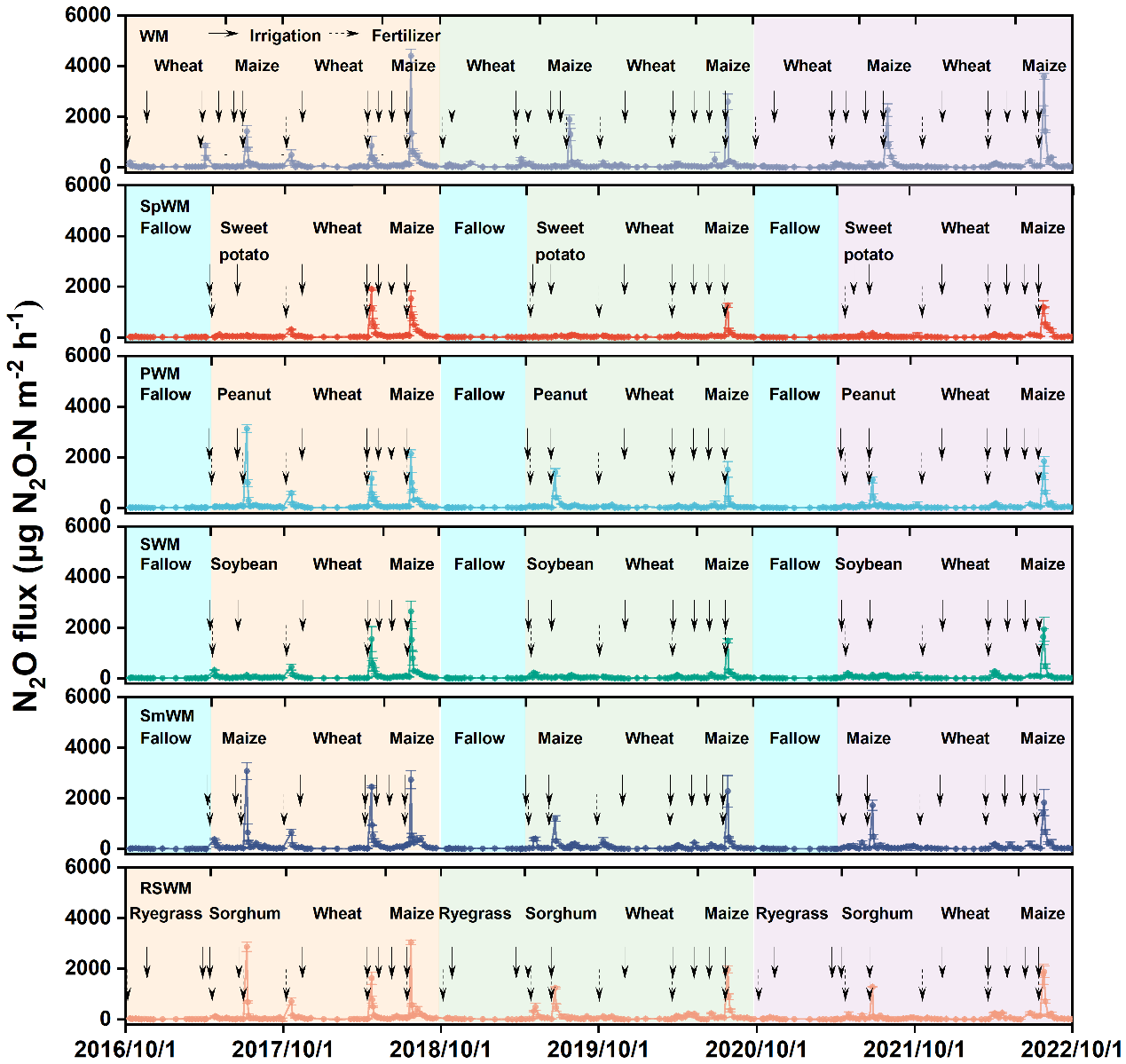
**Fig. S1** Time series of at least weekly measured soil N_2_O fluxes as observed for the different crop rotations. Values are means (*n*=3) ± SE for each measurement. Error bar for each dot is the standard deviation of three replicates. Treatment abbreviations: WM: winter wheat–summer maize (Control); SpWM: sweet potato→winter wheat–summer maize rotation; PWM: peanut→winter wheat–summer maize rotation, SWM: soybean→winter wheat–summer maize rotation, SmWM: spring maize→winter wheat–summer maize rotation, RSWM: ryegrass–sorghum→winter wheat–summer maize rotation.


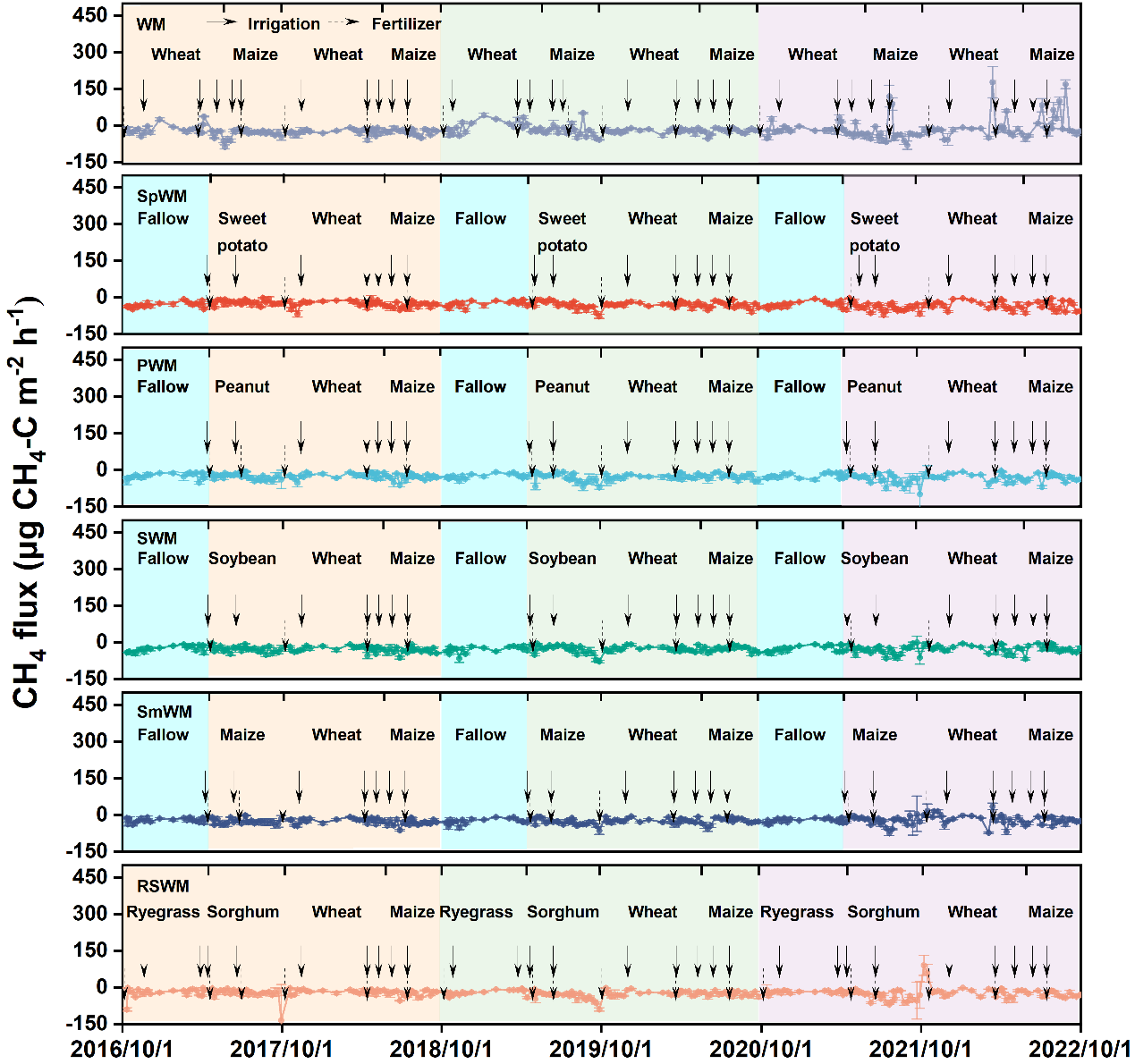
**Fig. S2** Time series of at least weekly measured soil CH_4_ fluxes as observed for the different crop rotations. Values are means (*n*=3) ± SE for each measurement. Error bar for each dot is the standard deviation of three replicates. Treatment abbreviations: WM: winter wheat–summer maize (Control); SpWM: sweet potato→winter wheat–summer maize rotation; PWM: peanut→winter wheat–summer maize rotation, SWM: soybean→winter wheat–summer maize rotation, SmWM: spring maize→winter wheat–summer maize rotation, RSWM: ryegrass–sorghum→winter wheat–summer maize rotation.


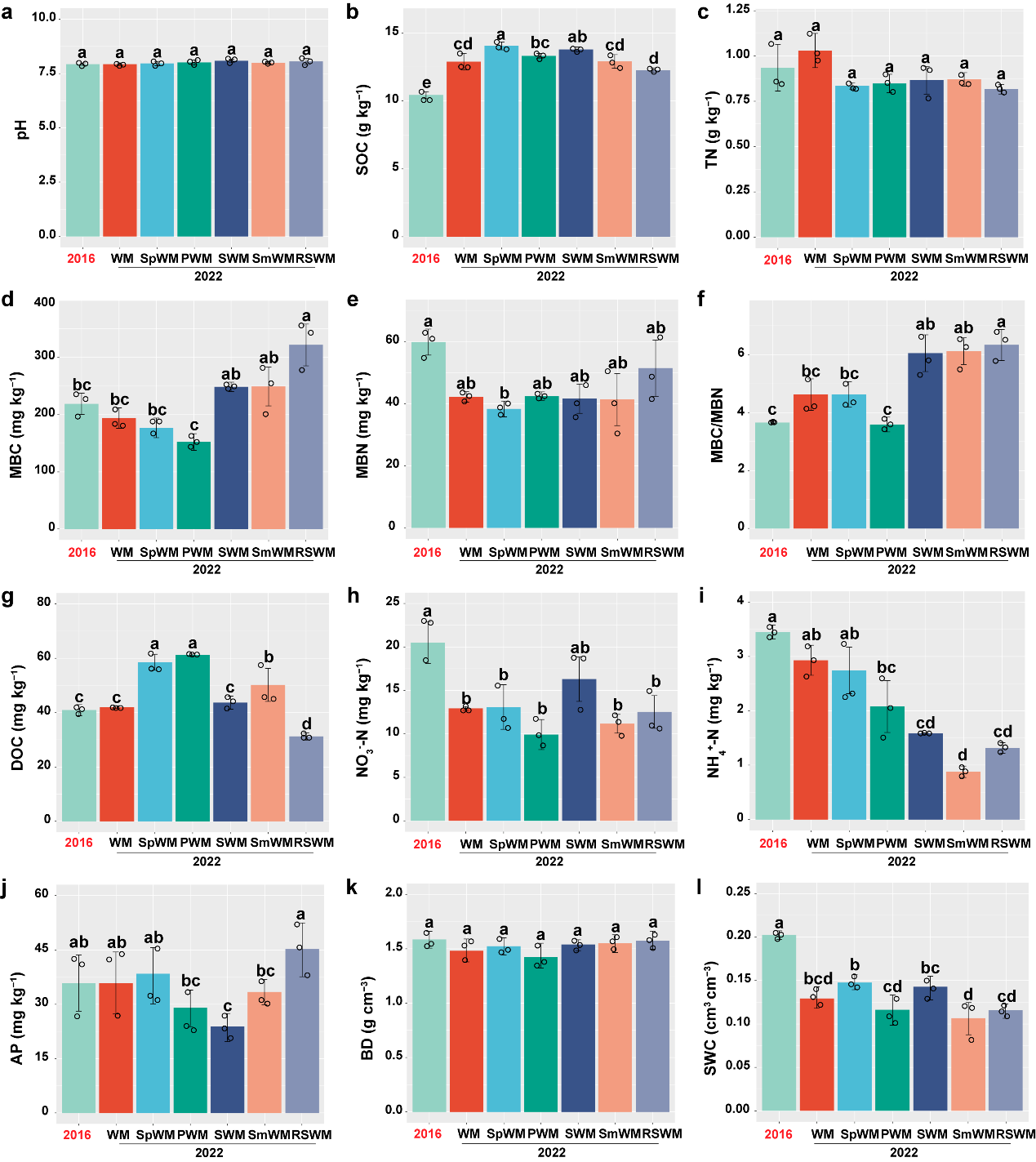


**Fig. S3** Variations in soil physicochemical and biological properties with different crop rotations in 2016 and 2022. Values are the mean of three replicates samples taken in October 2016 or October 2022. Different lowercase letters indicate significant differences between crop rotations for each indicator at *P* < 0.05, respectively. SOC: soil organic carbon, TN: total N, MBC: microbial biomass carbon, MBN: microbial biomass N, DOC: dissolved organic carbon, AP: available P, BD: bulk density, and SWC: soil water content. Treatment abbreviations: WM: winter wheat–summer maize (Control); SpWM: sweet potato→winter wheat–summer maize rotation; PWM: peanut→winter wheat–summer maize rotation, SWM: soybean→winter wheat–summer maize rotation, SmWM: spring maize→winter wheat–summer maize rotation, RSWM: ryegrass–sorghum→winter wheat–summer maize rotation.


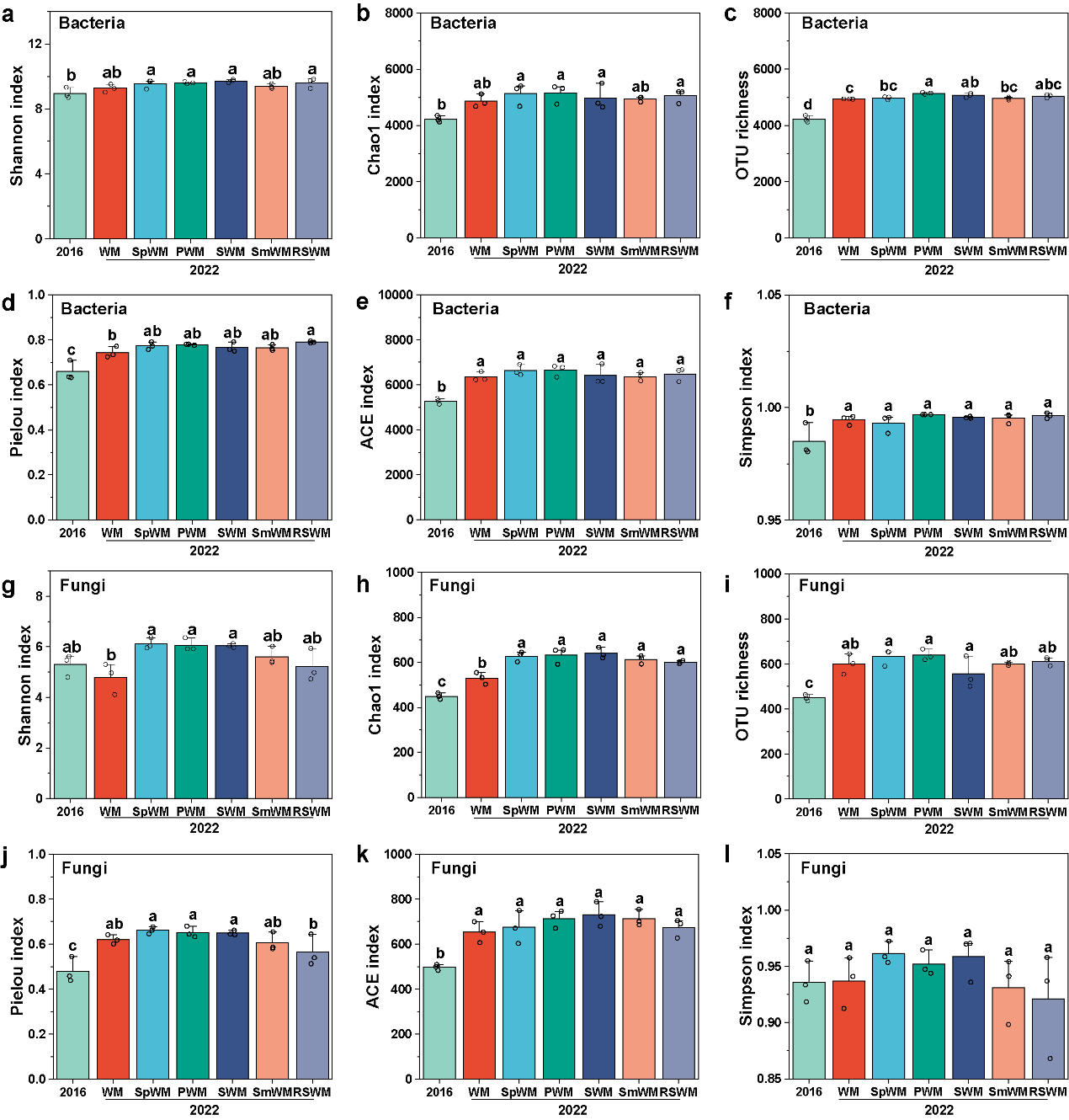
**Fig. S4** Changes in soil microbial biodiversity indexes between sampling dates in 2016 and 2022 following the implementation of six diversified crop rotations. (a–f): bacteria; (g–l): fungi. See SI Methods for the index calculations (Shannon Index, Chao 1, OTU Richness, ACE index, Pielou, and Simpson). Values are the mean of three replicates samples taken in October 2016 or October 2022. Different lowercase letters indicate significant differences between crop rotations for each indicator at *P*< 0.05, respectively. Treatment abbreviations: WM: winter wheat–summer maize (Control); SpWM: sweet potato→winter wheat–summer maize rotation; PWM: peanut→winter wheat–summer maize rotation, SWM: soybean→winter wheat–summer maize rotation, SmWM: spring maize→winter wheat–summer maize rotation, RSWM: ryegrass–sorghum→winter wheat–summer maize rotation.

**Supplementary Methods Figures**


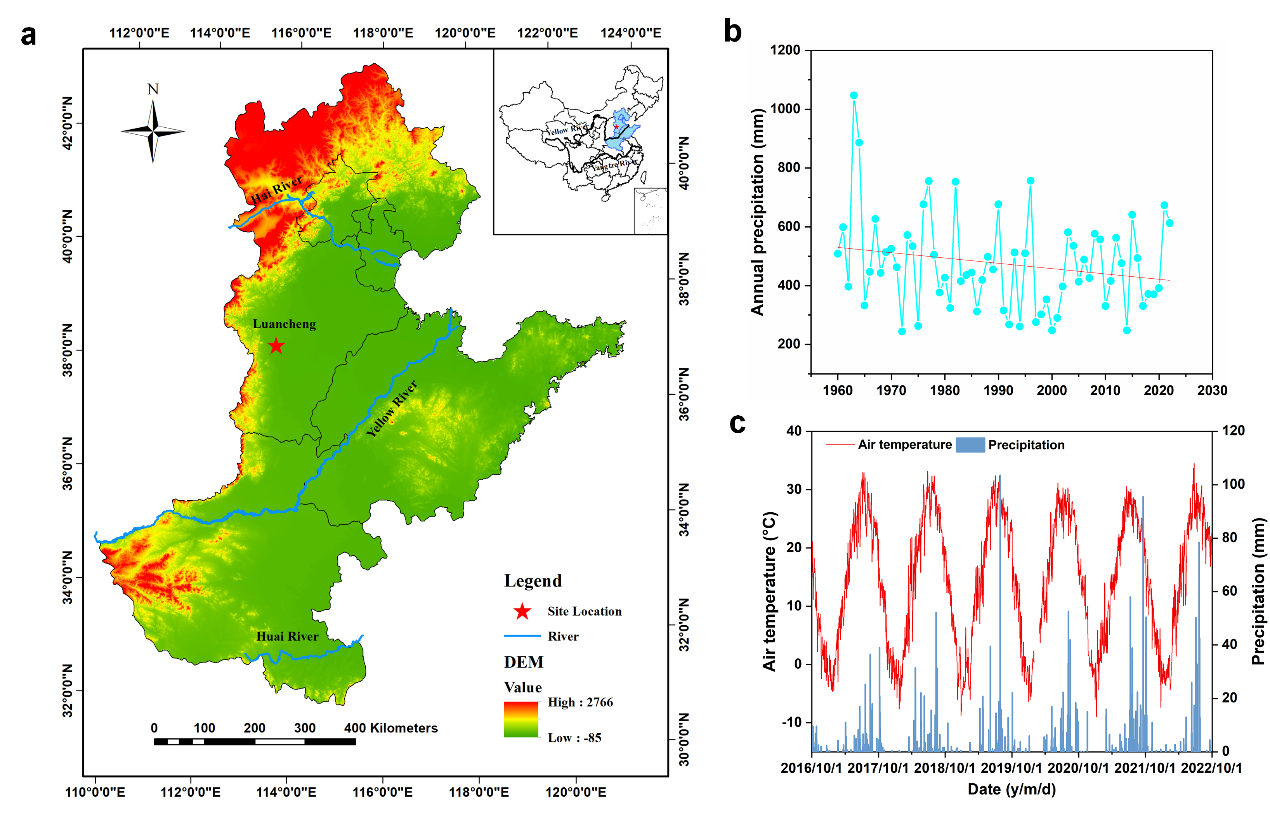


**Fig. S5** Location of Luancheng experimental station (a), annual precipitation from 1960 to 2022 (b), and monthly precipitation and average daily air temperature from 2016 to 2022 (c). (a) made using ArcGIS (Ver.10.5, ESRI Inc., CA, USA, 2016).


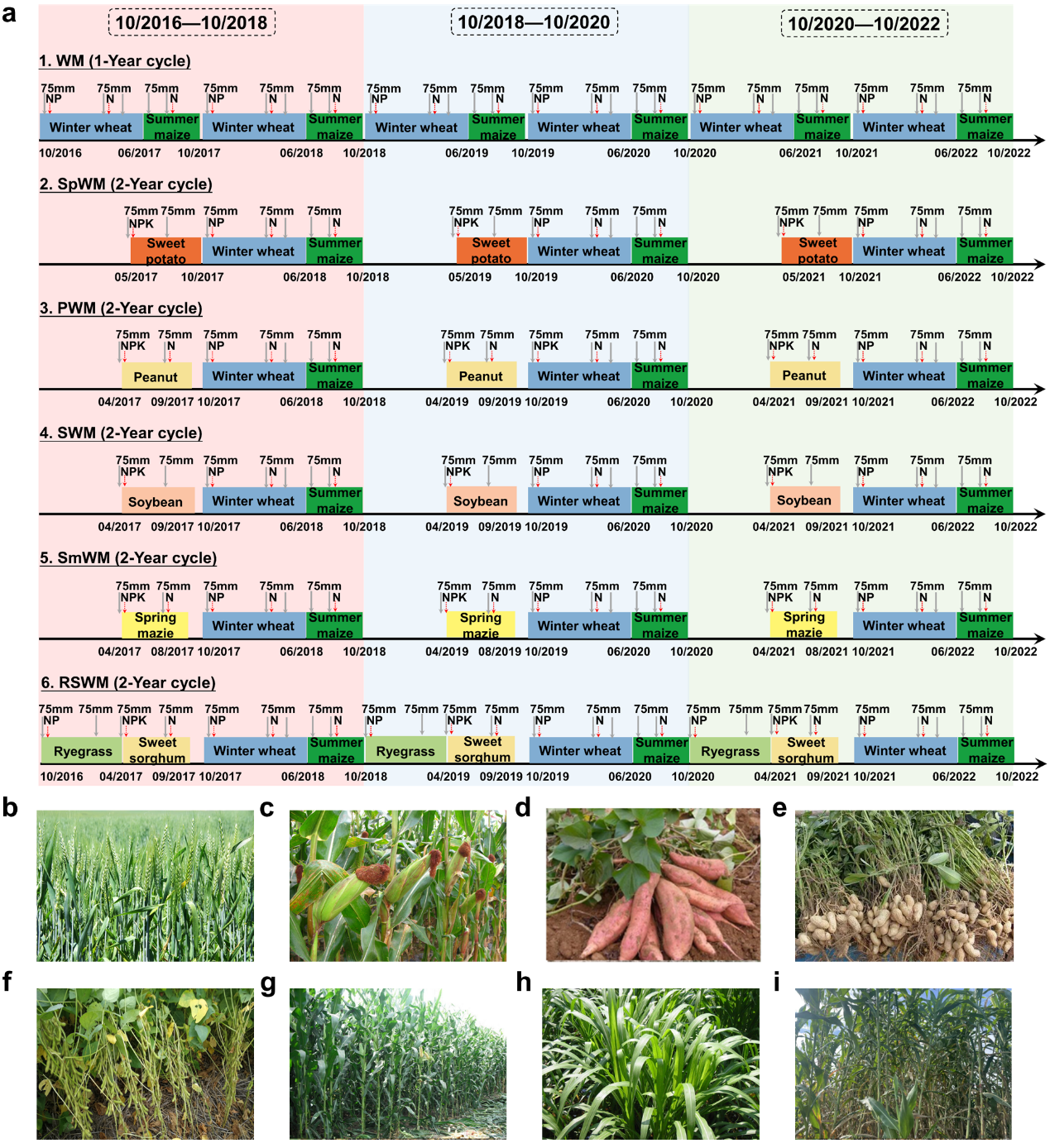
**Fig. S6** Graphical outline of cropping sequences and timing of field operations for the six investigated crop rotations (a). See Table S2 for dates, irrigation amounts, and fertilizer rates. Photos of winter wheat (b), summer maize (c), sweet potato (d), peanuts (e), soybean (f), spring maize (g), ryegrass (h), and sorghum (i).


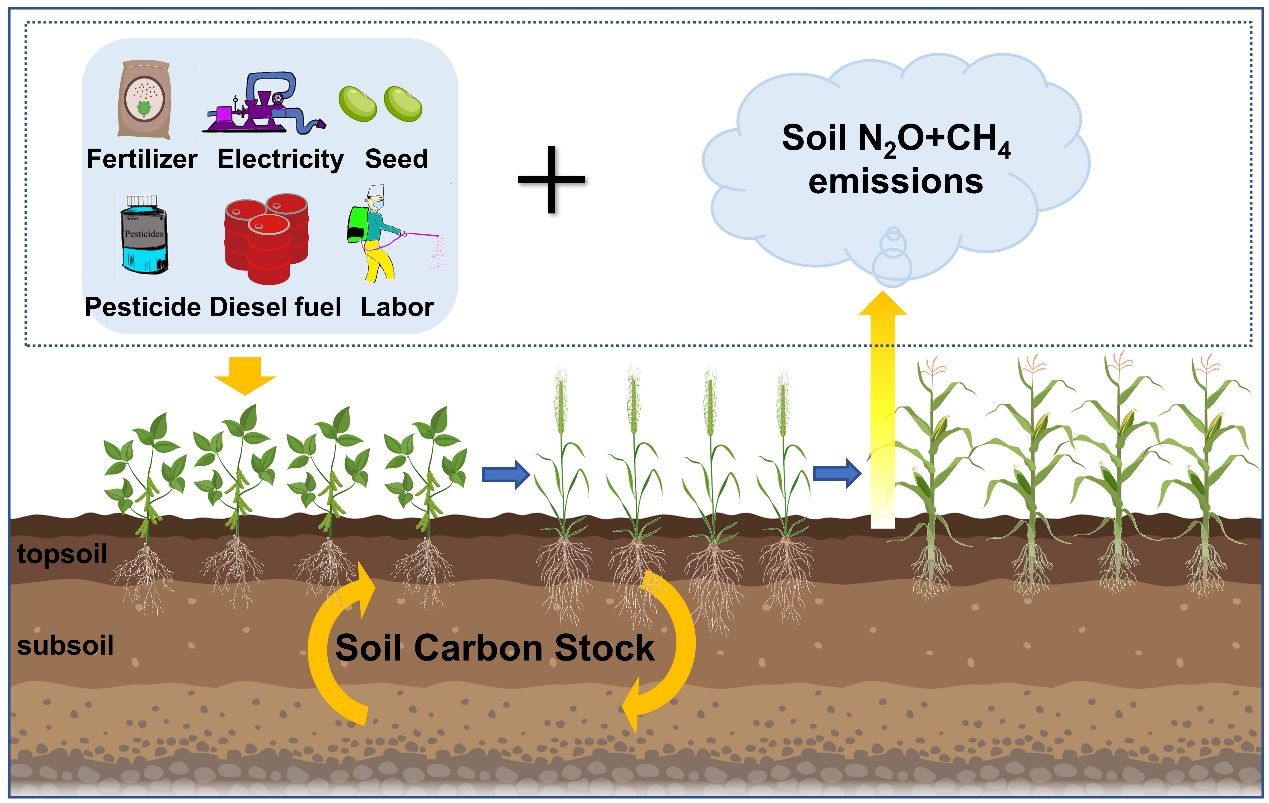


**Fig. S7** System boundary for calculating net greenhouse emissions in different crop rotations (small objects generated using BioRender (https://biorender.com/)).


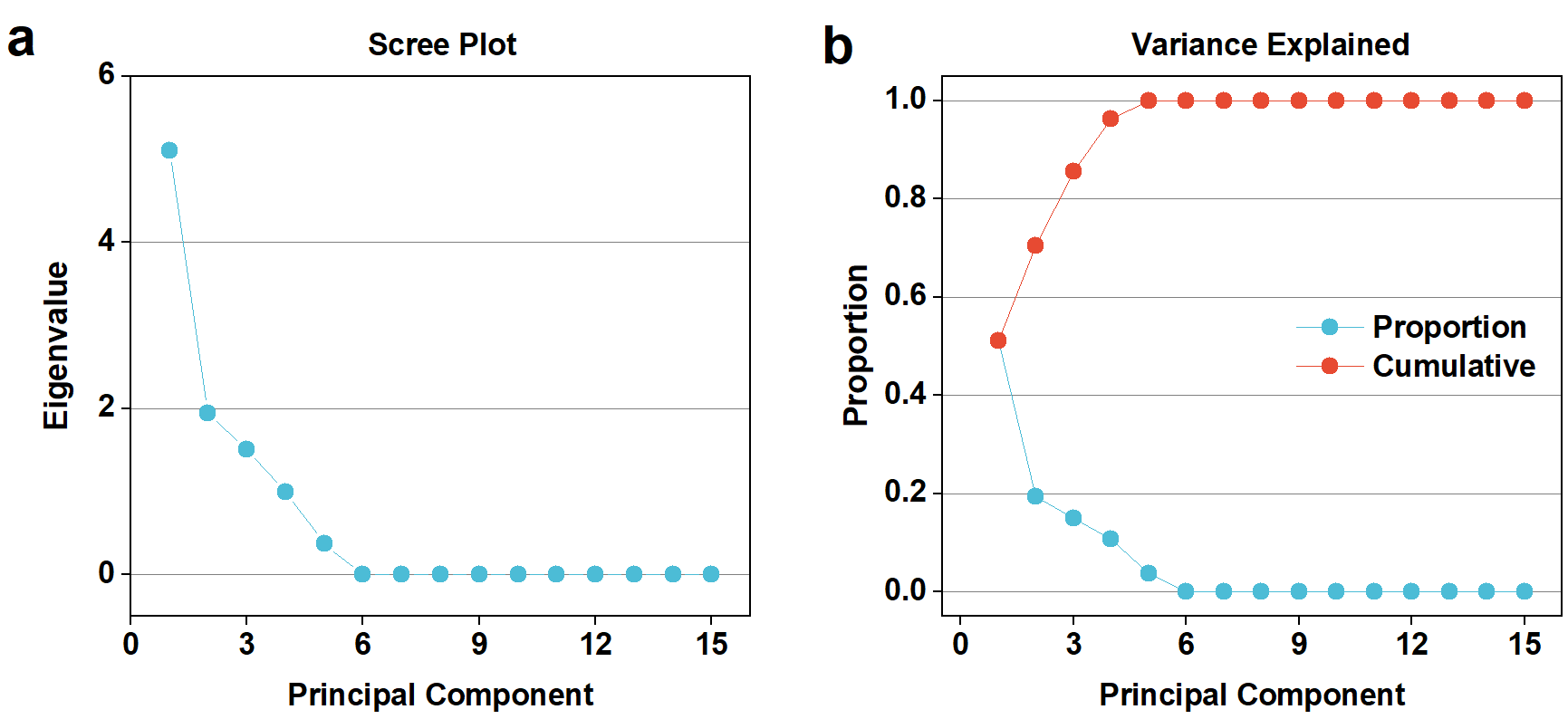


**Fig. S8** Scree plot of eigenvalues against the number of principal components (a, b) for soil indicators from six crop rotations.

**Table S1** Annual agricultural inputs for each crop in six crop rotations from 2016 to 2022

| Crop rotations | Crop |  | N (kg ha^–1^) | | P_2_O_5_  (kg ha^–1^) | K_2_O  (kg ha^–1^) | Irrigation (mm) | Diesel  (kg ha^–1^) | Electricity^a^  (kWh ha^–1^) | Pesticide^b^ (kg ha^–1^) | Seeds (kg ha^–1^) | Labor  (h ha^–1^) |
| --- | --- | --- | --- | --- | --- | --- | --- | --- | --- | --- | --- | --- |
|  |  | Total  N | Base N | Top  dressing |  |  |  |  |  |  |  |  |
| WM | Winter wheat | 310.2 | 118.5 | 191.7 | 299.0 | 0 | 225 | 319.2 | 900.5 | 8.4 | 80 | 201.8 |
|  | Summer maize | 283.7 | 0 | 283.7 | 0 | 0 | 150 | 279.1 | 600.3 | 4.8 | 72 | 22.5 |
| SpWM | Sweet potato | 27 | 27.0 | 0 | 69.0 | 150.0 | 150 | 139.5 | 600.3 | 3.8 | 68 | 375.0 |
|  | Winter wheat | 310.2 | 118.5 | 191.7 | 299.0 | 0 | 225 | 319.2 | 900.5 | 8.4 | 80 | 201.8 |
|  | Summer maize | 283.7 | 0 | 283.7 | 0 | 0 | 150 | 279.1 | 600.3 | 4.8 | 72 | 22.5 |
| PWM | Peanut | 156.3 | 75.0 | 81.3 | 191.7 | 83.3 | 150 | 139.5 | 600.3 | 0.7 | 68 | 333.3 |
|  | Winter wheat | 310.2 | 118.5 | 191.7 | 299.0 | 0 | 225 | 319.2 | 900.5 | 8.4 | 80 | 201.8 |
|  | Summer maize | 283.7 | 0 | 283.7 | 0 | 0 | 150 | 279.1 | 600.3 | 4.8 | 72 | 22.5 |
| SWM | Soybean | 151.8 | 151.8 | 0 | 172.5 | 150.0 | 150 | 139.5 | 600.3 | 0.8 | 68 | 45.0 |
|  | Winter wheat | 310.2 | 118.5 | 191.7 | 299.0 | 0 | 225 | 319.2 | 900.5 | 8.4 | 80 | 201.8 |
|  | Summer maize | 283.7 | 0 | 283.7 | 0 | 0 | 150 | 279.1 | 600.3 | 4.8 | 72 | 22.5 |
| SmWM | Spring maize | 224.5 | 134.8 | 89.7 | 89.7 | 91.7 | 150 | 40.1 | 600.3 | 0.9 | 72 | 22.5 |
|  | Winter wheat | 310.2 | 118.5 | 191.7 | 299.0 | 0 | 225 | 179.7 | 900.5 | 8.4 | 80 | 201.8 |
|  | Summer maize | 283.7 | 0 | 283.7 | 0 | 0 | 150 | 279.1 | 600.3 | 4.8 | 72 | 22.5 |
| RSWM | Ryegrass | 118.5 | 118.5 | 0 | 299.0 | 0 | 150 | 139.5 | 600.3 | 0 | 64 | 100.0 |
|  | Sweet sorghum | 224.5 | 134.8 | 89.7 | 89.7 | 91.7 | 150 | 139.5 | 600.3 | 2.4 | 72 | 22.5 |
|  | Winter wheat | 310.2 | 118.5 | 191.7 | 299.0 | 0 | 225 | 319.2 | 900.5 | 8.4 | 80 | 201.8 |
|  | Summer maize | 283.7 | 0 | 283.7 | 0 | 0 | 150 | 279.1 | 600.3 | 4.8 | 72 | 22.5 |

^a^ Electricity for irrigation is calculated as crop irrigation consumption (m^3^ ha^–1^) × unit electricity consumption (kWh m^–3^), and the unit for electricity consumption is 0.4 kWh m^–3^.

^b^ Pesticide values are the sum of the active ingredients for herbicides, insecticides, and fungicides.

**Table S2** CPI, price, and cost per hectare of diverse crops from 2016 to 2022

| Items | 2017 | 2018 | 2019 | 2020 | 2021 | 2022 |
| --- | --- | --- | --- | --- | --- | --- |
| CPI^a^ | 637.5 | 650.9 | 669.8 | 686.5 | 692.7 | 692.7 |
| Price^b^ (Yuan kg^–1^) | | | | | | |
| Sweet potato | 2.00 |  | 2.00 |  | 2.00 |  |
| Soybean | 6.03 |  | 6.02 |  | 7.28 |  |
| Peanut | 6.80 |  | 6.80 |  | 6.80 |  |
| Spring maize | 1.90 |  | 2.01 |  | 2.79 |  |
| Ryegrass | 1.41 |  | 1.41 |  | 1.41 |  |
| Sweet sorghum | 0.21 |  | 0.21 |  | 0.21 |  |
| Winter wheat | 2.53 | 2.58 | 2.57 | 2.66 | 2.83 | 2.83 |
| Summer maize | 1.90 | 1.99 | 2.01 | 2.24 | 2.79 | 2.79 |
| Cost^c^ (Yuan ha^–1^) | | | | | | |
| Sweet potato | 10371 |  | 12039 |  | 11483 |  |
| Soybean | 5193 |  | 5900 |  | 5998 |  |
| Peanut | 8168 |  | 8393 |  | 8416 |  |
| Spring maize | 4865 |  | 5105 |  | 4963 |  |
| Ryegrass | 4889 |  | 4889 |  | 4889 |  |
| Sweet sorghum | 4370 |  | 4400 |  | 4415 |  |
| Winter wheat | 7445 | 7497 | 7617 | 7774 | 8179 | 8432 |
| Summer maize | 4559 | 4859 | 4784 | 4859 | 4882 | 4687 |

^a^ CPI is the national Consumer Price Index from the China Yearbook of Agriculture Price Survey.

^b^ Ryegrass and sweet sorghum[^6^](#_ENREF_6); other crops from the China Yearbook of Agriculture Price Survey.

^c^ Cost is based on the actual field situation, including seed, fertilizer, electricity for irrigation, and fuel cost.

**Table S3** Nutritional content of different crops as reported in the literature

| Crops | Protein content (%) | N content (%) | P content (%) | K content (%) | Fat content (%) | Magnesium (mg kg^–1^) | Calcium (mg kg^–1^) | Sodium (mg kg^–1^) | Iron (mg kg^–1^) | Zinc (mg kg^–1^) | Vitamin A (mg kg^–1^) | Thiamin (mg kg^–1^) | Riboflavin (mg kg^–1^) | Folate (mg kg^–1^) | Niacin (mg kg^–1^) | Vitamin B6 (mg kg^–1^) | Vitamin E (mg kg^–1^) |
| --- | --- | --- | --- | --- | --- | --- | --- | --- | --- | --- | --- | --- | --- | --- | --- | --- | --- |
| Sweet potato (fresh weight) | 2 | 1 | 0 | 1 | 0 | 250 | 300 | 550 | 6 | 3 | 100 | 1 | 1 | 110 | 6 | 2 | 3 |
| Peanut | 26 | 4 | 0 | 1 | 49 | 1680 | 920 | 180 | 46 | 33 | 0 | 6 | 1 | 2400 | 121 | 3 | 83 |
| Soybean | 41 | 6 | 1 | 2 | 20 | 2800 | 2770 | 20 | 157 | 49 | 10 | 9 | 9 | 3750 | 16 | 4 | 9 |
| Spring maize | 8 | 2 | 0 | 0 | 5 | 1270 | 70 | 350 | 27 | 22 | 110 | 4 | 2 | 190 | 36 | 6 | 5 |
| Ryegrass | 12 | 2 | 0 | 2 | 0 | 772 | 1112 | 0 | 0 | 7 | 0 | 0 | 0 | 0 | 0 | 0 | 0 |
| Sweet sorghum | 4 | 1 | 1 | 2 | 0 | 0 | 0 | 0 | 0 | 0 | 0 | 0 | 0 | 0 | 0 | 0 | 0 |
| Winter wheat | 14 | 2 | 0 | 0 | 2 | 930 | 320 | 20 | 46 | 33 | 0 | 4 | 1 | 380 | 44 | 4 | 10 |
| Summer maize | 10 | 2 | 0 | 0 | 5 | 1270 | 70 | 350 | 27 | 22 | 110 | 4 | 2 | 190 | 36 | 6 | 5 |

Note: Protein content sources[^1^](#_ENREF_1)^,^[^7-12^](#_ENREF_7). Other nutritional parameters were supplied by Associate Professor Jeroen Groot from Wageningen University and Groot and Yang[^1^](#_ENREF_1).

**Table S4** Emission coefficients of carbon dioxide equivalent for agricultural inputs as reported in the literature

| Item | Unit | Value | Reference |
| --- | --- | --- | --- |
| Chemical N fertilizer | kg CO_2_-eq kg^–1^ | 8.30 | [^13^](#_ENREF_13) |
| P_2_O_5_ | kg CO_2_-eq kg^–1^ | 1.14 | [^14^](#_ENREF_14) |
| K_2_O | kg CO_2_-eq kg^–1^ | 0.66 | [^14^](#_ENREF_14) |
| Diesel fuel | kg CO_2_-eq kg^–1^ | 3.32 | [^14^](#_ENREF_14) |
| Electricity | kg CO_2_-eq kWh^–1^ | 0.92 | [^14^](#_ENREF_14) |
| Pesticide | kg CO_2_-eq kg^–1^ | 6.58 | [^14^](#_ENREF_14) |
| Labor | kg CO_2_-eq person^–1^ d^–1^ | 0.86 | [^14^](#_ENREF_14) |
| Seeds | | | |
| Winter wheat | kg CO_2_-eq kg^–1^ | 0.40 | [^15^](#_ENREF_15) |
| Maize | kg CO_2_-eq kg^–1^ | 2.10 | [^15^](#_ENREF_15) |
| Peanut | kg CO_2_-eq kg^–1^ | 0.92 | [^15^](#_ENREF_15) |
| Ryegrass | kg CO_2_-eq kg^–1^ | 1.98 | [^15^](#_ENREF_15) |
| Soybean | kg CO_2_-eq kg^–1^ | 0.92 | [^15^](#_ENREF_15) |
| Sweet sorghum | kg CO_2_-eq kg^–1^ | 3.15 | [^15^](#_ENREF_15) |
| Sweet potato | kg CO_2_-eq kg^–1^ | 0.10 | [^16^](#_ENREF_16) |

**Table S5** Weighting factors based on principal component analysis of eigen-vectors for the different soil indicators to calculate the soil health score for different crop rotations

|  | Soil attributes | Units | Weight factors |
| --- | --- | --- | --- |
| Physical | Bulk density | g cm^–3^ | 0.106 |
|  | Soil water content | % | 0.083 |
| Chemical | Soil pH |  | 0.079 |
|  | Total nitrogen | g kg^–1^ | 0.114 |
|  | Soil organic carbon | g kg^–1^ | 0.111 |
|  | Dissolved organic carbon | mg kg^–1^ | 0.102 |
|  | Nitrate nitrogen | mg kg^–1^ | 0.098 |
|  | Available Phosphorus | mg kg^–1^ | 0.103 |
| Biological | Microbial biomass carbon | mg kg^–1^ | 0.102 |
|  | Microbial biomass nitrogen | mg kg^–1^ | 0.102 |

**References:**

1. Groot, J. C. J. & Yang, X. L. Trade-offs in the design of sustainable cropping systems at a regional level: A case study on the North China Plain. *FASE* **9**, 14 (2022).

2. Zhao, X. M. *et al.* A new technique for determining micronutrient nutritional quality in fruits and vegetables based on the entropy weight method and fuzzy recognition method. *Foods* **11**, 3844 (2022).

3. Spellerberg, I. F. & Fedor, P. J. A tribute to Claude Shannon (1916–2001) and a plea for more rigorous use of species richness, species diversity and the ‘Shannon–Wiener’ Index. *Global Ecol. Biogeogr.* **12**, 177-179 (2003).

4. Kim, B. R. *et al.* Deciphering Diversity Indices for a Better Understanding of Microbial Communities. *J. Microbiol. Biotechnol.* **27**, 2089-2093 (2017) (eng).

5. Zou, Y. *et al.* Impacts of Intercropped Maize Ecological Shading on Tea Foliar and Functional Components, Insect Pest Diversity and Soil Microbes. *Plants* **11**, 1883 (2022).

6. Yang, X. L., Sun, B. B., Gao, W. S., Chen, Y. Q. & Sui, P. Carbon footprints of grain-, forage-, and energy-based cropping systems in the North China plain. *Int. J. Life Cycle Assess.* **24**, 371-385 (2019).

7. Baldinger, L., Baumung, R., Zollitsch, W. & Knaus, W. F. Italian ryegrass silage in winter feeding of organic dairy cows: forage intake, milk yield and composition. *J. Sci. Food Agric.* **91**, 435-442 (2011).

8. FAO. *FAO/INFOODS Food Composition Database for Biodiversity Version 4.0 ‐ BioFoodComp4.0*, (Food and Agriculture Organization of The United Nations, Rome, Italy, 2017).

9. Hou, M. *et al.* QTL mapping and interaction analysis of seed protein content and oil content in soybean. *Scientia Agr. Sin.* **47**, 2680-2689 (2014).

10. Qi, L. J. *et al.* Analysis of wheat protein quality in main wheat producing areas of China from 2004 to 2011. *Scientia Agr. Sin.* **45**, 4242-4251 (2012) (in Chinese).

11. Zhang, X. Y. *et al.* Application of near-infrared spectroscopy technology to analyze protein content in single kernel maize seed. *J. Chin. Agr. Univ.* **22**, 25-31 (2017) (in Chinese).

12. Zhang, Y., Zhao, W. J., Chang, Y. H., Shao, Y. F. & Yun, W. H. Selection and cultivation techniques of energy purposed sweet sorghum Jintianza 3. *J. Shanxi Agr. Sci.* **42**, 816-818 (2014) (in Chinese).

13. Zhang, W. F. *et al.* New technologies reduce greenhouse gas emissions from nitrogenous fertilizer in China. *Proc. Natl. Acad. Sci. U.S.A.* **110**, 8375-8380 (2013).

14. Liu, X. H. *et al.* The missteps, improvement and application of carbon footprint methodology in farmland ecosystems with the case study of analyzing the carbon efficiency of China’s intensive farming. *Chin. J. Agric. Resour. Reg. Plan.* **34**, 1-11 (2013).

15. West, T. O. & Marland, G. A synthesis of carbon sequestration, carbon emissions, and net carbon flux in agriculture: comparing tillage practices in the United States. *Agric. Ecosyst. Environ.* **91**, 217-232 (2002).

16. Xu, Z. Y., Xu, W. J., Zhang, Z. H., Yang, Q. Y. & Meng, F. J. Measurement and evaluation of carbon emission for different types of carbohydrate-rich foods in china. *Chem. Eng. Trans.* **61**, 409-414 (2017).
