# Supplementary material for: Diversifying crop rotation increases food production, reduces net greenhouse gas emissions and improves soil health
Source: Nat Commun. 2024 Jan 3;15:198. doi: 10.1038/s41467-023-44464-9 (PMC10764956; doi:10.1038/s41467-023-44464-9)
Supplement: Supplementary file 4 — Reporting Summary [file 41467_2023_44464_MOESM4_ESM.pdf]

nature portfolio

Corresponding author(s): Xiaolin Yang,Taisheng Du,Xiaotang Ju,Yantai Gan

Last updated by author(s): Dec 10, 2023

Reporting Summary

Nature Portfolio wishes to improve the reproducibility of the work that we publish. This form provides structure for consistency and transparency in reporting. For further information on Nature Portfolio policies, see our Editorial Policies and the Editorial Policy Checklist.

Statistics

For all statistical analyses, confirm that the following items are present in the figure legend, table legend, main text, or Methods section.

n/a	Confirmed	
☐	☒	The exact sample size (*n*) for each experimental group/condition, given as a discrete number and unit of measurement
☐	☒	A statement on whether measurements were taken from distinct samples or whether the same sample was measured repeatedly
☐	☒	The statistical test(s) used AND whether they are one- or two-sided<br>*Only common tests should be described solely by name; describe more complex techniques in the Methods section.*
☒	☐	A description of all covariates tested
☐	☒	A description of any assumptions or corrections, such as tests of normality and adjustment for multiple comparisons
☐	☒	A full description of the statistical parameters including central tendency (e.g. means) or other basic estimates (e.g. regression coefficient) AND variation (e.g. standard deviation) or associated estimates of uncertainty (e.g. confidence intervals)
☐	☒	For null hypothesis testing, the test statistic (e.g. *F*, *t*, *r*) with confidence intervals, effect sizes, degrees of freedom and *P* value noted<br>*Give P values as exact values whenever suitable.*
☒	☐	For Bayesian analysis, information on the choice of priors and Markov chain Monte Carlo settings
☐	☒	For hierarchical and complex designs, identification of the appropriate level for tests and full reporting of outcomes
☐	☒	Estimates of effect sizes (e.g. Cohen's *d*, Pearson's *r*), indicating how they were calculated

Our web collection on statistics for biologists contains articles on many of the points above.

Software and code

Policy information about availability of computer code

Data collection	We did not use any other data collection softwares in this study. The dataset of crop yield, biomass, soil N2O flux, CH4 flux, soil samples for soil properties and soil bacterial and fungal community were collected from the 6-year field experiment by the authors and their teams. Crop price and national Consumer Price Index were download as excel files from China Yearbook of Agricultural Price Survey, National Bureau of Statistics of China.
Data analysis	Statistical analyses were conducted in SPSS version 21 software (IBM's Statistical Product and Service Solutions) and R version 4.3.178. The Kolmogorov-Smirnov test was used for testing normal distribution, followed by an analysis of variance (ANOVA) to determine the statistical significance of differences between mean values. Post-hoc comparisons were conducted using the least significant difference (LSD) test at the 5% probability level. The correlation matrix of indicators for each rotation was calculated with Pearson's and visualized using the Performance Analytics package in R 4.3.1 and R Studio 1.8.0. Principal component analysis (PCA) was used to calculate the eigenvalues and classify each soil indicator in each rotation using the vegan package, visualized using the ggplot2 package in R 4.3.1. The α-diversity indexes of the soil microbial community were calculated using the vegan and picante packages, visualized using R 4.3.1. A radar map illustrating the performance of the multiple objective analysis was produced to assess the various functions of each rotation, visualized using ggradar packages. A Nightingale Rose Chart was used to assess the functions of each crop rotation, visualized using ggplot2 package. Customer codes scripts are available at https://doi.org/10.6084/m9.figshare.24793563.

For manuscripts utilizing custom algorithms or software that are central to the research but not yet described in published literature, software must be made available to editors and reviewers. We strongly encourage code deposition in a community repository (e.g. GitHub). See the Nature Portfolio guidelines for submitting code & software for further information.

Data

Policy information about availability of data

All manuscripts must include a data availability statement. This statement should provide the following information, where applicable:

- Accession codes, unique identifiers, or web links for publicly available datasets
- A description of any restrictions on data availability
- For clinical datasets or third party data, please ensure that the statement adheres to our policy

The dataset generated in this study has been deposited in the database under accession code on Figshare at hhttps://doi.org/10.6084/m9.figshare.24793563. Source data are provided with this paper.

Research involving human participants, their data, or biological material

Policy information about studies with human participants or human data. See also policy information about sex, gender (identity/presentation), and sexual orientation and race, ethnicity and racism.

Reporting on sex and gender	No
Reporting on race, ethnicity, or other socially relevant groupings	No
Population characteristics	No
Recruitment	No
Ethics oversight	No

Note that full information on the approval of the study protocol must also be provided in the manuscript.

Field-specific reporting

Please select the one below that is the best fit for your research. If you are not sure, read the appropriate sections before making your selection.

☐ Life sciences ☐ Behavioural & social sciences ☒ Ecological, evolutionary & environmental sciences

For a reference copy of the document with all sections, see nature.com/documents/nr-reporting-summary-flat.pdf

Ecological, evolutionary & environmental sciences study design

All studies must disclose on these points even when the disclosure is negative.

Study description	This experiment was a randomized complete block design with six treatments. It is a single-factor experiment. The six treatments were randomized in each of three replicates, resulting in 18 experimental units. The six treatments are six different rotations, involving sweet potato, soybean, peanut, ryegrass, sorghum and spring maize as previous crops before cereal crops and the conventional winter wheat-summer maize rotation; the crops in the six rotations were repeated on their respective plots each year from October 2016 to October 2022.
Research sample	Plants , soil, and soil microbial samples were taken from each treatment in each of three replications each year from October 2016 to October 2022. The rationale is to allow for the verification and validation of study findings.
Sampling strategy	All field studies require multiple replications. In this study, we used three replications for each treatment; the sample size (i.e., three replicates) was determined based on the sample size principle of statistical requirements (SPSS version 21). The three replication data were used in the analyses of ANOVA, PCA, RDA and Pearson's Correlation.<br><br>Each crop was harvested at maturity, with biomass, crop yield, and yield components measured of each plot. Soil greenhouse gas emissions (N2O and CH4 fluxes) were measured using the static chamber method and were collected about once every 7 days from sowing to harvest, with more frequent measurements (every 3 days) after N fertilizer application and precipitation events. Gas samples were analyzed using a gas chromatograph. Each item of crop cultivation input was recorded for indirect greenhouse gas emission calculations. Soil samples (0–20 cm depth) were collected at the beginning of the experiment (October 2016) and at the end of summer maize harvest in October 2022 to measure soil physicochemical properties: including soil pH; bulk density; soil water content; total nitrogen (TN); dissolved organic carbon (DOC); soil nitrate-N (NO3−-N) and ammonium-N (NH4+-N); available phosphorus (AP); and microbial biomass carbon and nitrogen (MBC and MBN, respectively). Soil organic carbon (SOC) concentrations in the 0–90 cm soil layer (0–10, 10–20, 20–30, 30–50, 50–70, and 70–90 cm increments) were measured after each crop harvest from October 2016 to October 2022, in samples collected by auger at five random locations per plot. Soil samples of 0-20 cm were also collected before the start of the experiment (October 2016) and during the final summer maize harvest (October 2022) for DNA sequencing and microbial community composition and diversity measurements. Detailed in Methods and supplementary materials.
Data collection	All the data were measured and collected using equipment and tools from authors' laboratories and recorded by authors using

	Microsoft Excel.
Timing and spatial scale	The database were collected from each of three replicates in each of six years of field experiments (from October 2016 to October 2022). The rationale is to provide the appropriate timing and spatial scales.
Data exclusions	No data was excluded from the analysis.
Reproducibility	The field experiment can be replicated or performed independently any time. The study is fully reproducible using the methods and procedures detailed in the manuscript and supplementary material.
Randomization	Six rotation treatments were randomized in each of the three blocks.
Blinding	Blinding is not relevant to our study

Did the study involve field work? ☒ Yes ☐ No

Field work, collection and transport

Field conditions	The field experiment was established in October 2016. The experimental area experiences a warm, temperate zone, semi-humid, monsoon climate with a frost-free period of 200 days. The annual mean air temperature was 14.70 ± 1.0℃ over the last 20 years, and the annual average precipitation was 472 ± 161 mm over the last 62 years. The experimental field has loam soil with sandy loam in the surface layers, light/medium loam at 40–80 cm depth, and light clay below 80 cm. The 0–10 cm soil layer has pH 7.6 ± 0.15, 240 ± 42 µS cm-1 EC, 1.49 ± 0.07 g cm-3 bulk density, 0.42 ± 0.03 cm3 cm-3 field capacity, 0.10 ± 0.02 cm3 cm-3 wilting point, 1.06 ± 0.14 g kg-1 total N content, 11.5 ± 0.38 g kg-1 SOC concentration, 9.33 ± 3.12 mg kg-1 available phosphorous, and 109.6 ± 24 mg kg-1 available potassium.
Location	The field experiment was conducted at the Luancheng Agro-Ecosystem Station of the Chinese Academy of Sciences (37°50′ N, 114° 40′ E; altitude, 50.1 m) in Luancheng County, Hebei Province, which represents the agricultural production and climate conditions of the North China Plain.
Access & import/export	All plant and soil samples were collected and measured in a responsible manner and in compliance with local, national and international laws.
Disturbance	Not aware of any disturbance involved in this study.

Reporting for specific materials, systems and methods

We require information from authors about some types of materials, experimental systems and methods used in many studies. Here, indicate whether each material, system or method listed is relevant to your study. If you are not sure if a list item applies to your research, read the appropriate section before selecting a response.

Materials & experimental systems

n/a	Involved in the study
☒	☐ Antibodies
☒	☐ Eukaryotic cell lines
☒	☐ Palaeontology and archaeology
☒	☐ Animals and other organisms
☒	☐ Clinical data
☒	☐ Dual use research of concern
☒	☐ Plants

Methods

n/a	Involved in the study
☒	☐ ChIP-seq
☒	☐ Flow cytometry
☒	☐ MRI-based neuroimaging

Plants

Seed stocks	The experiment comprised eight crops, including three: cereal crops [winter wheat (cv. Kenong 2009), summer maize (cv. Jundan 20), and spring maize (cv. Jundan 20)], two; legume crops [soybean (cv. Shidou 12) and peanut (cv. Jihua 4)], and three; cash/forage crops: [sweet potato (cv. Shangshu 19), ryegrass (cv. Dongmu 70), and sorghum (cv. Jintianza 3)]
Novel plant genotypes	No
Authentication	No
